# Food Safety Knowledge and Hygienic Practices among Different Groups of Restaurants in Muscat, Oman

**DOI:** 10.1155/2020/8872981

**Published:** 2020-12-19

**Authors:** Maryam Al-Ghazali, Ismail Al-Bulushi, Lyutha Al-Subhi, Mohammad Shafiur Rahman, Amani Al-Rawahi

**Affiliations:** ^1^Al Joudah Food Tech lab, Seeb, Oman; ^2^Department of Food Science and Nutrition, College of Agricultural and Marine Sciences, Sultan Qaboos University, Seeb, Oman; ^3^Purple Swans Company, Seeb, Oman

## Abstract

Food safety is vital to human beings as well as to the food industry. Therefore, knowledge and hygiene practice of food safety among food handlers are particularly important. Evaluation of food safety knowledge and hygienic practices among 18 restaurants in three different regions (i.e., districts) in the Governorate of Muscat was performed. In order to determine the quality level of restaurants, grouping (i.e., Region 1, Region 2, and Region 3; e.g., Group I, Group II, and Group III) was adopted from the regulations and assigned by the municipality based on the number of complaints against the restaurants. A questionnaire was designed to assess the knowledge of hygiene, food poisoning, food handling, cooking, and local regulations/rules of the food handlers in selected restaurants. The food handlers in the Region 1 restaurants showed significantly higher (*p* < 0.05) overall knowledge (58.6%) in food safety as compared to Region 2 (52.1%) and Region 3 (53.2%). Overall knowledge of food handlers in Group I restaurants was significantly higher (*p* < 0.05) (64.4%) as compared to Group II (53.1%) and Group III (48.1%). The hygiene practices in Group I restaurants were significantly higher (*p* < 0.05) than those in Groups II and III. A small but significant inverse association (*r*^2^ = −0.38) between total knowledge scores and hygiene practices was found. In conclusion, higher knowledge in the field is associated with better hygiene practices, and these are more likely to prevent food poisoning originating from restaurants. We recommend implementing specialized education courses and workshops for the food handlers as a requirement before embarking on service to decrease the risks of foodborne diseases.

## 1. Introduction

Food poisoning is considered as one of the most food-related disease that takes lives, hospitalizes people, and loses many communities incomes. The burden of this problem varies and could affect all population in the developed and developing countries. For example, among 600 million of foodborne diseases reported worldwide in 2010, 550 million of the cases were caused by the common bacterial pathogens such as Campylobacter spp., Vibrio cholerae, Shigella spp., enteropathogenic *Escherichia coli*, and enterohaemorrhagic *Escherichia coli* [1]. Irrespective of the types of causative agents of foodborne diseases, denying the access of the causative agent, increasing the food safety knowledge of the food handlers, and implementing good hygienic practices remain the most effective strategies to control and minimize the burdens of foodborne diseases in any community. In fact, restaurants have been commonly found as important places for microbial pathogens access to the food, which could cause food poisoning outbreaks.

Therefore, in many food poisoning outbreaks in the developed counties, restaurants have received special interest among other possible access for pathogens and were widely found to be associated with many poisoning outbreaks in many developed countries [2–5]. However, since the food poisoning outbreaks are not medically and microbiologically investigated in many developing counties, the role of the restaurants as an access of causative pathogens remains unclear. The impact of food safety knowledge among food handlers to minimize the risks of foodborne diseases was evaluated in some studies [6, 7]. For instance, in South Africa, attendance of food safety course was found to have a significant effect on some practices, such as wearing gloves and washing hands [6].

Many food poisoning outbreaks were attributed by unhygienic practices in different countries, such as Italy, China, and United Sates of America [8–10]. For instance, the first Salmonella enterica subspecies enterica serovar Berta food poisoning outbreak in Italy was explained by poor hygienic practices in the kitchen and food preparation areas [8]. The hygiene status in the restaurants and in food handling services was evaluated by enumeration of the typical contamination and hygiene indicator, namely, Enterobacteriaceae, in some studies [11–13]. In fact, Enterobacteriaceae count of 4 log CFU/g or higher in foods such as sashimi was considered as unsatisfactory level [13].

[14] presented a cross-sectional study on the determinants of food hygiene knowledge among youths in premier university based in Kuala Lumpur city, Malaysia. As in any community, some food poisoning outbreaks were reported in Oman [1, 15]. These outbreaks occurred in different food service areas such as restaurants and parties. Despite the concerned numbers of outbreaks, which occasionally occur especially during summer, information related to food safety knowledge among the food handlers and food hygiene practices in food service premises is limited in Oman. Therefore, this study is aimed at evaluating the level of food safety knowledge among the food handlers and the hygienic status in some restaurants in order to elucidate the role of possible factors involved in food poisoning outbreaks in Oman as well as at evaluating the safe food handling status in the food premises such as restaurants.

## 2. Materials and Methods

### 2.1. Questionnaire Design for Assessing Knowledge

A questionnaire was developed based on previous studies [15–20]. Some questions were based on food safety standards of Food and Agriculture Organization (FAO), World Health Organization (WHO), and International Organization for Standardization (ISO) [21, 22]. The questionnaire composed of two sections: demographic characteristics and knowledge in different parameters (hygiene, food poisoning, food handling, cooking, and knowledge in municipality rules including food safety and hygiene practices). The knowledge questions had different response options: “Yes/No,” “true/false,” “multiple choices,” and “short answers.” The questionnaire was developed in English then was translated into Arabic, Urdu, and Hindi. For knowledge questions, each correct answer was given 1 point, while incorrect responses, do not know, and unanswered questions were given 0 point following the procedure of [23]. The scores were normalized as percentage. Knowledge scores below 50% were categorized as poor knowledge.

### 2.2. Restaurants and Samples

Data was collected from February to July 2016. In total, the study included 18 restaurants from 3 regions in the Governorate of Muscat, Oman (i.e., Region 1, Region 2, and Region 3). Restaurants were classified into two groups. The first classification was performed based on geographical locations, and it is denoted by Regions 1, 2, and 3. The second classification was based on the number of complaints issued by Muscat Municipality, and these were considered as Group I (i.e., low complaints), Group II (i.e., medium complaints), and Group III (i.e., high complaints) as categorized from the record of the Muscat Municipality. Total 54 food handlers responded to the questionnaire. The questionnaire was self-administered, and it took about 20-30 minutes to complete. Two restaurants were visited per week, and three food handlers were selected from each restaurant.

In order to determine hygienic practice among food handlers, a total 162 swab samples (54 food handlers' hand, 54 chopping boards, and 54 knives) were collected 20 minutes before food preparation. During the process of swabbing, the hygienic procedure was maintained using gloves, masks, sanitizers, and sterilizing aluminum. Swab samples were collected as per the procedures of [18, 22, 24, 25]. The swab samples were immediately kept in a cool box with crushed ice that was used to maintain the box temperature close to 0°C (1-5°C). After swab collection, the cool boxes were sent to the laboratory within 2-4 hours as per the method of [26]. Muscat Municipality provided assistance in relation to the information on statistical data and municipality regulations.

### 2.3. Bacterial Enumeration

Total Aerobic Bacterial Count (TABC) and *Enterobacteriaceae* (ENT) were enumerated on Standard Plate Count Agar (SPCA) and Violet Red Bile Glucose Agar (VRBGA), respectively. Agar plates were incubated for 24 hours at 37°C following the procedures of previous studies [18, 24, 27, 28]. Bacterial counts were reported as log_10_ cfu/cm^2^.

### 2.4. Statistical Analyses

The statistical analysis of data was performed using the correlation and regression analysis. Data was analyzed by SPSS version 20. Findings with a *p* value < 0.05 were considered statistically significant. Frequencies, percentage, and mean were applied to describe the characteristic of the respondents and hygienic practices. One-way ANOVA was applied to assess differences in knowledge and hygiene practices within regions and groups. Post hoc multiple comparisons within groups were assessed by Fisher's Least Significant Difference (LSD). Pearson's correlation was used to test the association between food handler's knowledge scores and hygiene practices.

## 3. Results and Discussion

### 3.1. Food Handlers' Knowledge

#### 3.1.1. Food Handlers' Knowledge

Table 1 illustrates the demographic characteristics of 54 food handlers from the 18 restaurants under study. Our results showed that 54 food handlers washed their hands after using the toilet, in accordance with those of [29] who reported that 95% of the food handlers in restaurants in Vienna washed their hand after using the toilet. This could be due to the compliance of food handlers to the rules set by the companies in relation to enforcing or emphasizing washing hand after using toilet in both countries. In addition, regular training attended by food handlers could have influenced their adherence to the above rules. Most of food handlers in our study wore gloves (81.5%) and did not wear rings and accessories (96.3%) while cooking. These findings are in accordance with those of [20], who observed that food handlers in campus restaurants, in a Malaysian university, wore gloves, covered mouth, and did not wear jewelry during food preparation.

Most of food handlers (70.4%) did not have an idea about Good Manufacturing Practice (GMP) and Hazard Analysis Critical Control Point (HACCP) due to lack of formal training (Table 2). However, only 24.6% of the participants showed knowledge on the ppm levels of chlorine (Table 2), which must be used within 100-200 ppm in washing vegetables [30]. About 50% of the food handlers are smokers; however, 80.1% of them reported never smoking inside the preparation areas. As smoking is banned in the preparation area according to the WHO guidelines [22], the remaining (20%) of those reported, smoking could be a possible risk factor for the safety. The incidence of smoking in the preparation areas in the current study was similar to that reported by [31], who observed that 16.1% of the food handlers smoked in food preparation areas in Saudi Arabia.

**Figure 1:**
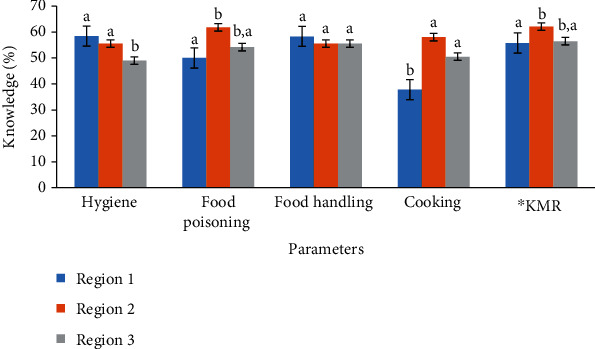
Food safety and hygiene knowledge of food handlers in restaurants among different regions in Muscat (different letters show significant difference, *p* < 0.05). ^∗^KMR: knowledge of the municipality rules.

Figure 1 illustrates the percentage of knowledge in food safety and hygiene among food handlers working in the different regions in Muscat Government (i.e., Region 1, Region 2, and Region 3). LSD multiple comparison results showed that Region 3 had significantly (*p* < 0.05) lower mean knowledge (49.0%) in hygiene than Regions 1 and 2. As suggested by [23], such a level of knowledge is considered poor since it is lower than 50.0%. Similarly, [32] observed a low level of hygiene knowledge (43.4%) when 47.8% of their staff did not get enough training on food safety and hygiene. There was not a significant difference between the food handlers' knowledge in Regions 1 (58.5%) and 2 (55.6%) (*p* > 0.05) as evaluated by Fisher's LSD. The poor knowledge of food handlers in Region 3 could be linked to the large number of complaints it received due to a variety of violations. Furthermore, 8.6% of food handlers had less than secondary school, and 50% of them had secondary school, and they had lack of knowledge on food hygiene parameter in Region 3.

Food handlers in the three regions had relatively good knowledge of food poisoning, food handling, and of the municipality rules (i.e., scores ≥ 50.0%). However, food handlers in Region 2 had significantly (*p* < 0.05) lower level of knowledge than those in Region 1 in terms of food poisoning, municipality rules on food safety, hygiene practices, and the availability of labor card in the restaurants (Figure 1). There was no significant difference with Region 3 (*p* > 0.05) (Figure 1). Moreover, there was no significant difference in the level of knowledge of food handling among the different regions (*p* > 0.05), which could have been influenced by the level of education among the participants where majority attained secondary school or less (Table 1). In the literature, [33] observed knowledge level of 40.0% in the food preparation and handling, while [31] observed 65.8% of knowledge of food poisoning domain which is supported by the fact that their staff were excellent in practices towards cross contamination and personal hygiene.

Food handlers in Region 2 had significantly (*p* < 0.05) higher level of knowledge of safe cooking (37.8%) than their counterparts in Region 1 (58.1%) and Region 3 (50.5%) (Figure 1). Similarly, [32] observed that knowledge of safe cooking was 45.5% in their study due to a lack of knowledge about the basics of food hygiene. However, we found a significant difference (*p* < 0.05) in the level of food handler's knowledge on cooking (e.g., cooking temperature and time and cooling methods) between Region 1 and Region 3. Overall, the food handlers in Region 1 had significantly higher score (58.6%) on food safety compared to Region 2 (52.1%) and Region 3 (53.2%) (*p* < 0.05). The food handlers in Region 1 had more working experience (i.e., 20-28 years) as compared to Region 2 and Region 3; thus, it could be another reason of better performance.

#### 3.1.2. Knowledge Based on Groups of the Restaurants

**Figure 2:**
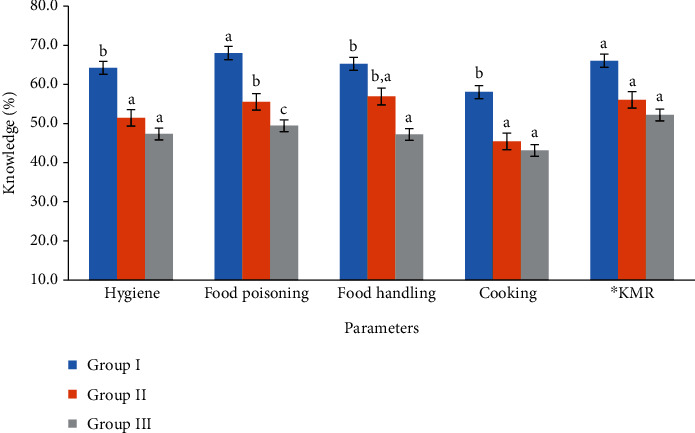
Food safety and hygiene knowledge of food handlers among all groups at Muscat restaurants (different letters show significant difference, *p* < 0.05). ^∗^KMR: knowledge of the municipality rules.

Figure 2 illustrates that food handlers in Group I (low complaints) had significantly higher level of knowledge (hygiene 64.3%, food poisoning 68.1%, and cooking 58.1%) than the other two groups. However, there was no significant difference in knowledge between the food handlers in Groups II and III considering hygiene, cooking, food handling, and their knowledge in municipality rules on food safety, hygiene practices, and the availability of labor card in the restaurants (*p* > 0.05) (Figure 2). However, there was a significant difference between the food handlers' knowledge in Group II and Group III considering food poisoning (*p* < 0.05). Overall knowledge for Group I was considered good as compared to the other groups since it was more than 50.0% as suggested by [23]. However, food handlers in Group III possessed poor knowledge considering all parameters except their knowledge on the municipality rules. Low average score of the food handlers in Group III could be the reason of the large number of complaints it received. It was mentioned by the municipality that they made more visits to the Group III. Similarly, [18] observed that food handlers had poor knowledge on food poisoning (42.6%) and safe storage (i.e., food handling) (47.2%) and cooking (49.0%) as in Group III in the current study.

Food handlers in the Group I had significantly higher level of knowledge on food handling (65.3%) as compared to the Group III (47.2%). Oppositely, [33] observed poor knowledge level of 40.0% on the food preparation and handling. In addition, there was no a significant difference (*p* > 0.05) among groups considering knowledge of the municipality rules because most of them received the same information and rules from the municipality. The reasons for higher scores of the food handlers in Group I could be due to that 33.4% of food handlers had postsecondary education compared to Group II (11.1%) and Group III (0%). In addition, 15.6% of food handlers in the Group I attended more training courses on food safety and hygiene on HACCP, compared to the other groups. Moreover, food handlers in Group I were fluent in English, and their activities were constantly monitored by a specific Person in Charge (PIC). Similarly, [18] observed that food handlers in food premises possessed more knowledge in hygiene (79.9%) as compared to other parameters. The reasons for their higher value in hygiene knowledge as compared to this study could be due to their communication skills (i.e., English) and their experience. They reported that food handlers with less than 10-year experience had an average score of 61.5% compared to those with more than 10 years (65.8%). In addition, food handlers who received training had better food safety information than those who did not get training. However, most of the food handlers in Group III were poor in English skill (83.3%) and had low training in the food handling (79.6%).

### 3.2. Hygiene Practices

#### 3.2.1. Hygiene Practices in Different Regions

Table 3 shows that the hygiene practices, based on the microbial quality and handling, in Region 3 were significantly lower (*p* < 0.05) than the hygiene practices in Regions 2 and 1 restaurants. The reasons for low hygiene practices in Region 3 could be due to low level of education and insufficient knowledge of food handlers in food safety as compared to the Regions 2 and 1. For example, the level of food handlers' knowledge score on food safety was 52.1% in Region 3 as compared to 58.6 and 53.2% for Region 1 and Region 3, respectively.

Regions 1 and 2 showed a higher hygienic status than Region 3, which could be due to the significant difference in knowledge of hygiene among the regions as shown in Figure 1. It was observed, from the field, that the restaurants in Region 3 used unclean cleaning sponge as compared to other regions. Moreover, for all regions, TABC for all categories of contaminations (i.e., food handlers, chopping boards, and knives) was above the standard 2.0 log cfu/cm^2^ recommendation by WHO [22]. In Region 1 and Region 2, microbial loads of knives and chopping boards were significantly lower (TABC) (i.e., 2.1 log cfu/cm^2^ and 2.7 log cfu/cm^2^) than knives (i.e., 2.9 log cfu/cm^2^) and chopping boards (3.0 log cfu/cm^2^) in Region 3 (*p* < 0.05). Similarly, [27] reported higher TABC of the hands of food handlers in the delicatessen than the standard 2 log cfu/cm^2^. The authors attributed the findings to an increased handling of high-risk foods. In contrast, [34] reported, for restaurants in Italy, lower TABC for hands and food contact surfaces (i.e., knives and cutting boards) than the standard of 2.0 log cfu/cm^2^ [22]. They pointed that their staff had frequently attended health education courses. Some plausible reasons for the high microbial (TABC) load in Region 3 in our study are poor standards and malpractices such as the use of the same chopping board for both vegetables and chicken (2.0% of food handlers) and improper hand washing (66.7% of the food handlers). It is clear from the results that restaurants in Region 2 had the lowest microbial loads due to higher knowledge of food handlers in the food poisoning, cooking, and knowledge of the municipality rules.

Table 4 illustrates the hygiene practices among restaurants in different regions, based on sources of cross contamination. Results showed that ENT counts of food handlers (1.7 log cfu/cm^2^), chopping boards (1.4 log cfu/cm^2^), and knives (1.7 log cfu/cm^2^) in Region 3 restaurants were significantly higher than those in Region 1 and Region 2 (*p* < 0.05), which suggests of a poor hygiene practices. There was no significant difference between Region 2 and Region 1 (*p* > 0.05). The reasons for low hygiene practices in Region 3 could be due to the low education level and insufficient food safety knowledge of food handlers as compared to the Region 2 and Region 1. In Region 3, ENT counts, for all categories of contamination, were higher than the recommended standard of 0-1.3 log cfu/cm^2^ [22]. ENT counts for Region 2 and Region 1, for all categories of contamination, were within the recommended standard. Moreover, [26] reported that restaurants in Iowa State had significantly low (*p* < 0.05) hygiene practices, since ENT count on the cutting boards was found to be 3.0 log cfu/cm^2^ as compared to standard 0-1.3 log cfu/cm^2^ [22]. The ENT load in the current study was lower than that was found by [26] but higher than that by [35]. Similarly, [34] observed high hygiene standard in the restaurants in Italy considering ENT for cross contamination (i.e., hand and food contact surface: 0-1 log cfu/cm^2^).

#### 3.2.2. Hygienic Practices among Different Groups of Restaurants

Table 5 illustrates that the hygiene practices in Group I restaurant (i.e., TABC count: food handlers' hands, 2.3 log cfu/cm^2^; chopping boards, 2.3 log cfu/cm^2^; and knives, 1.8 log cfu/cm^2^) were significantly higher (*p* < 0.05) than the hygiene practices in restaurants in Group II and Group III. The reasons for the high hygiene practices in the Group I could be due to the high education level of food handlers (i.e., postsecondary: Group I, 33.4%; Group II, 11.1%; and Group III, 0.0%) and good knowledge around food handling (i.e., 65.3%). Cooking scores of Group I, Group II, and Group III were 58.1, 45.5, and 43.1%, respectively. Furthermore, food handlers in Group I controlled the temperature during cooking and thawing properly as compared with the other groups (i.e., Groups II and III).

In all groups, TABC for all categories was higher than the standard of 2 log cfu/cm^2^ recommended by WHO [22], except for knives (1.8 log cfu/cm^2^) in Group I. The reasons for observed low TABC for knives could be due to the use of clean sponges during the washing process of knives based on FDA and FAO recommendations, reducing thus the chances of cross contamination.

Table 5 illustrates that the microbial load of knives (TABC: 1.8 log cfu/cm2) of Group I were significantly (*p* < 0.05) lower than that of Group II (2.7 log cfu/cm2) and Group III (2.5 log cfu/cm^2^), whereas the microbial load (TABC) on knives in Group II was higher than in Group III restaurants. The reasons for low microbial (TABC) load in Group I could be due to high care in washing of kitchen tools, high education level of food handlers, and training courses taken by the food handlers. Studying the handling and microbial quality in Ghana restaurants, [36] reported TABC count for chopping boards was higher than 3.0 log cfu/cm^2^; the standard is 2 log cfu/cm^2^. They linked these high numbers to the lack of personal hygiene practices and food handlers' knowledge. They therefore recommended enforcing high hygienic condition in their kitchen facility. Similarly, [25] found high TABC on knives as compared to standards (<2 log cfu/cm^2^) in selected Indian restaurants. They attributed this finding to the extremely poor hygiene practices of their staff.

Table 6 illustrates that Group I restaurants had the best hygiene practice with undetectable levels of ENT in all samples. Untraceable levels of ENT in this group could be due to good personal hygiene. There was a significant difference between the hygiene practices in Group II and Group III restaurants (*p* < 0.05). Group III had significantly better hygiene practices reflected by lower ENT load for chopping boards (2.1 log cfu/cm^2^) and knives (1.9 log cfu/cm^2^) (*p* < 0.05) than Group II, whereas Group II had significantly lower ENT load than Group III considering food handlers (*p* < 0.05).

In Group II, ENT for all categories of contamination was higher than the standard of 0-1.3 log cfu/cm^2^ (Table 6), whereas the microbial loads on chopping boards and knives in Group III were within the standard. Lack of personal hygiene and monitoring could be possible contributors to the high microbial load (ENT) in Group II [27]. [27] used ENT as hygiene indicators and observed low hygiene standard in the South African restaurants (hand: 1.5-1.8 log cfu/cm2). They attributed this to improper handling of different categories of raw foods as result of the high ENT on the hands of the food handlers. In contrast, [34] observed high hygiene standard (low microbial load) in the Italian restaurants with ENT < 1.3 log cfu/cm^2^.

### 3.3. Correlation between Knowledge and Hygiene Practices

**Figure 3:**
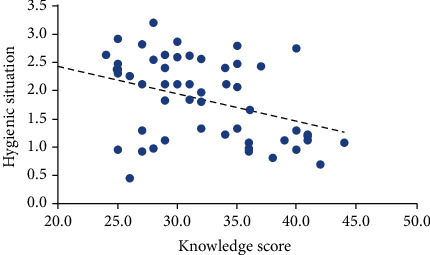
The correlation between knowledge and hygiene practice at selected restaurants in Muscat Governorate.

Figure 3 and Table 7 illustrate the correlation between knowledge among food handlers and hygiene practices by Pearson correlation based on the sum of ENT and TABC (i.e., total microbial count for both ENT and TABC) in the selected restaurant in Muscat. The association between knowledge and hygiene practices (*r =* -0.38) was significant (*p* < 0.05). The negative correlations indicated that higher knowledge score was related to lower microbial contaminations. The low correlation of knowledge and practices indicates that other factors rather than knowledge, such as following food regulations and moral of the food handlers, also played a role. [25] also observed negative relationship between knowledge and hygiene practices in restaurants in Vadodara Gujarat where only low group restaurants were considered. [37] also observed that food handlers' knowledge did not have a strong effect on hygiene practices (i.e., *r* = −0.20 and *p* = 0.04). Not only higher knowledge but also positive attitudes toward food safety did not always result in positive change in food handling practices. However, practices improved with the duration of employment (*r* = 0.40, *p* < 0.001).

## 4. Conclusion

The food handlers' hygienic knowledge and practices in Region 1 were significantly higher than other regions. Among groups, food handlers in Group I restaurants had significantly higher level of knowledge on hygiene, food poisoning, and cooking compared to Group II and Group III. This study showed that knowledge had significantly improved the hygienic practices (*p* < 0.05 and *r* = −0.38); however, the correlation indicated that there are other factors beyond the knowledge. Therefore, future studies need to be done on factors, such as attitude, motivation, and moral related to food hygiene practices and knowledge in order to improve the quality of food offered to the public by different levels of restaurants.

## Figures and Tables

**Table 1:** Personal information of the food handlers including age, gender, educational level, and experience (*n* = 54).

Characteristics of the food handlers	%
Age	
21-30	44.4
31-40	46.3
41-50	3.7
>50	5.6

Gender	
Female	3.7
Male	96.3

Education level	
Below secondary school	8.6
Secondary	50.0
Postsecondary	7.5

Courses and training attended	
None	79.6
HACCP	9.2
Food safety	16.7
Hygiene and sanitation program	9.2

Years of experience in food handling	
1-5	58.5
6–12	32.5
20-28	9.0

Responsibility in the restaurant	
Chef	4.4
Cooking and meal preparation	82.6
Serving	26.1

**Table 2:** Food handlers' knowledge.

Food handlers' knowledge	%
Training course	70.4
HACCP & GMP	

Food handling	
ppm levels of chlorine	24.6

Food handler's habits	
Smoking	80 not smoke

**Table 3:** Microbial quality in restaurants among regions based on Total Aerobic Bacterial Count (log cfu/cm^2^).

Samples/region	Region 1	Region 2	Region 3
Food handler	2.7^a^ ± 0.7	2.5^a^ ± 0.9	2.8^a^ ± 0.6
Chopping board	2.7^a^ ± 0.6	2.7^a^ ± 0.9	3.0^b^ ± 0.8
Knife	2.1^a^ ± 0.9	2.1^a^ ± 1.1	2.9^b^ ± 0.7

Different letters in row show significant difference (*p* < 0.05) as analyzed by Fisher's LSD.

**Table 4:** Hygiene practices and cross contamination in restaurants among regions based on *Enterobacteriaceae* (log cfu/cm^2^).

Samples/region	Region 1	Region 2	Region 3
Food handlers	1.0^a^ ± 0.8	1.0^a^ ± 1.2	1.7^b^ ± 1.1
Chopping board	1.0^a^ ± 1.1	1.2^a^ ± 1.2	1.4^a^ ± 1.2
Knife	1.0^a^ ± 1.0	1.0^a^ ± 0.8	1.7^b^ ± 1.4

Different letters in row show significant difference (*p* < 0.05) as analyzed by Fisher's LSD.

**Table 5:** Hygiene practices and microbial quality in restaurants among groups based on Total Aerobic Bacterial Count (log cfu/cm^2^).

Samples/group	Group I	Group II	Group III
Food handler	2.3^b^ ± 0.7	2.7^a^ ± 0.7	3.0^a^ ± 0.7
Chopping board	2.3^b^ ± 0.8	2.9^a^ ± 0.6	3.2^a^ ± 0.7
Knife	1.8^b^ ± 1.1	2.7^a^ ± 1.0	2.5^a^ ± 0.7

Different letters in row show significant difference (*p* < 0.05) as analyzed by Fisher's LSD.

**Table 6:** Hygiene practices and cross contamination in restaurants among groups based on Enterobacteriaceae (log cfu/cm^2^).

Samples/group	Group I	Group II	Group III
Food handlers	ND	1.4^a^ ± 1.2	1.8^b^ ± 1.2
Chopping board	ND	2.1^a^ ± 1.2	1.2^b^ ± 1.1
Knife	ND	1.9^a^ ± 0.8	1.1^b^ ± 1.4

Different letters in row show significant difference (*p* < 0.05) as analyzed by Fisher's LSD. ND: not detectable.

**Table 7:** The correlation between knowledge and hygiene practices at selected restaurants in Muscat Governorate.

Correlations			
		Knowledge	Hygiene practices

Knowledge	Pearson correlation	1	-0.38^∗^
	*p* value		*p* < 0.05
	*N*	54	54

Hygiene practices	Pearson correlation	-0.38^∗^	1
	*p* value	*p* < 0.05	
	*N*	54	54

^∗^Correlation is significant.

## Data Availability

All the data used to support the findings of this study are from previously reported studies and datasets, which have been cited in this manuscript. Furthermore, the processed data will be provided upon request.

## References

[1] Food and Agriculture Organization of the United Nations (2020). Climate change unpacking the burden on food safety. *Food Safety and Quality Series*.

[2] Firestone M. J., Lee P., Hedberg C. W. (2020). Improving inclusion and exclusion criteria in foodborne illness outbreak investigations a case study. *Epidemiology and Infection*.

[3] Thirkell C. E., Sloan-Gardner T. S., Kaczmarek M. C., Polkinghorne B. (2019). An outbreak of Bacillus cereus toxin-mediated emetic and diarrhoeal syndromes at a restaurant in Canberra, Australia 2018. *Communicable Diseases Intelligence*.

[4] Wu G., Yuan Q., Wang L. (2018). Epidemiology of foodborne disease outbreaks from 2011 to 2016 in Shandong Province, China. *Medicine*.

[5] Venkat H., Matthews J., Lumadao P. (2018). Salmonella enterica serotype Javiana infections linked to a seafood restaurant in Maricopa County, Arizona, 2016. *Journal of Food Protection*.

[6] Nkhebenyane J. S., Lues R. (2020). The knowledge, attitude, and practices of food handlers in central South African hospices. *Food Science & Nutrition*.

[7] Toh P. S., Birchenough A. (2000). Food safety knowledge and attitudes: culture and environment impact on hawkers in Malaysia.: knowledge and attitudes are key attributes of concern in hawker foodhandling practices and outbreaks of food poisoning and their prevention. *Food Control*.

[8] di Giannatale E., Sacchini L., Persiani T., Alessiani A., Marotta F., Zilli K. (2012). First outbreak of food poisoning caused by Salmonella enterica subspecies enterica serovar Berta in Italy. *Letters in Applied Microbiology*.

[9] Liu Y., Tam Y. H., Yuan J. (2015). A foodborne outbreak of gastroenteritis caused by Vibrio parahaemolyticus and norovirus through non-seafood vehicle. *PLoS One*.

[10] Centers for Disease Control and Prevention (2019). Foodborne illness outbreaks at retail establishments National Environmental Assessment Reporting System, 16 state and local health departments, 2014–2016.

[11] Rosiane C. N., Elizandra M. S., Jackline F. B. . S. J. (2018). Good hygiene practices and microbiological contamination in commercial restaurants. *African Journal of Microbiology Research*.

[12] Tóth J. A., Szakmár K., Dunay A., Illés B. C., Bittsánszky A. (2018). Hygiene assessments of school kitchens based on the microbiological status of served food. *Acta Scientiarum Polonorum Technologia Alimentaria*.

[13] Miguéis S., Santos C., Saraiva C., Esteves A. (2015). Evaluation of ready to eat sashimi in northern Portugal restaurants. *Food Control*.

[14] Low W. Y., Jani R., Halim H. A., Alias A. A., Moy F. M. (2016). Determinants of food hygiene knowledge among youths: a cross-sectional online study. *Food Control*.

[15] Ministry of Health Report (2015). *Annual Health Report*.

[16] De Wit J., Rombouts F. (1992). Faecal micro-organisms on the hands of carriers: Escherichia coli as model for Salmonella. *International Journal of Hygiene and Environmental Medicine*.

[17] Farahat M. F., El-Shafie M. M., Waly M. I. (2015). Food safety knowledge and practices among Saudi women. *Food Control*.

[18] Osaili T. M., Abu Jamous D. O., Obeidat B. A., Bawadi H. A., Tayyem R. F., Subih H. S. (2013). Food safety knowledge among food workers in restaurants in Jordan. *Food Control*.

[19] Rahman M. M., Arif M. T., Bakar K., Tambi Z. (2012). Food safety knowledge, attitude and hygiene practices among the street food vendors in Northern Kuching city, Sarawak. *Borneo Science*.

[20] Abdullah Sani N., Siow O. N. (2014). Knowledge, attitudes and practices of food handlers on food safety in food service operations at the Universiti Kebangsaan Malaysia. *Food Control*.

[21] NIST institute (2014). Nebosh international general certification. Health, safety, environment, training and consulting. http://www.nistinstitute.com.

[22] World Health Organization (2009). WHO guidelines on hand hygiene and food contact surfaces in health care. *Report, First Global Patient Safety Challenge Clean Care Is Safer Care. Normal bacterial flora on hands*.

[23] Tan S. L., Bakar F. A., Abdul Karim M. S., Lee H. Y., Mahyudin N. A. (2013). Hand hygiene knowledge, attitudes and practices among food handlers at primary schools in Hulu Langat district, Selangor (Malaysia). *Food Control*.

[24] Public Health England (2015). *Identification of Enterobacteriaceae. Report, UK Standards for Microbiology Investigations*.

[25] Sheth M., Gupta A., Ambegaonkar T. (2011). Handlers’ hygiene practices in small restaurants of Vadodara. *Nutrition & Food Science*.

[26] Sneed J., Strohbehn C., Gilmore S. A., Mendonca A. (2004). Microbiological evaluation of foodservice contact surfaces in Iowa assisted- living facilities. *Journal of the American Dietetic Association*.

[27] Lues J., van Tonder I. (2007). The occurrence of indicator bacteria on hands and aprons of food handlers in the delicatessen sections of a retail group. *Food Control*.

[28] Maturin L., Peeler T. (2001). *Food Drug Administration. Report, Bacteriological Analytical Method. Total Aerobic Bacterial Count*.

[29] Pichler J., Ziegler J., Aldrian U., Allerberger F. (2014). Evaluating levels of knowledge on food safety among food handlers from restaurants and various catering businesses in Vienna, Austria 2011/2012. *Food Control*.

[30] NSW and Food Authority (2006). *Industry Guide to Developing a Food Safety Program. Report, Hospital and Aged Care*.

[31] Al-Shabib N. A., Mosilhey S. H., Husain F. M. (2016). Cross-sectional study on food safety knowledge, attitude and practices of male food handlers employed in restaurants of King Saud University, Saudi Arabia. *Food Control*.

[32] Baş M., Şafak Ersun A., Kıvanç G. (2006). The evaluation of food hygiene knowledge, attitudes, and practices of food handlers’ in food businesses in Turkey. *Food Control*.

[33] Rosmawati N., Manan W. (2015). Validity and reliability of food safety knowledge and practices questionnaire among food handlers. *Health and the Environment Journal*.

[34] Balzaretti C. M., Marzano M. A. (2013). Prevention of travel-related foodborne diseases: microbiological risk assessment of food handlers and ready-to-eat foods in northern Italy airport restaurants. *Food Control*.

[35] Sagoo S., Little C., Griffith C., Mitchell R. (2003). Study of cleaning standards and practices in food premises in the United Kingdom. *Communicable Disease and Public Health/PHLS*.

[36] Addo K. K., Mensah G. I., Bonsu C., Akyeh M. L. (2007). Food and its preparation conditions in hotels in Accra, Ghana: a concern for food safety. *African Journal of Food, Agriculture, Nutrition and Development*.

[37] Ansari-Lari M., Soodbakhsh S., Lakzadeh L. (2010). Knowledge, attitudes and practices of workers on food hygienic practices in meat processing plants in Fars, Iran. *Food Control*.

[38] Yardimci H., Hakli G., Cakiroglu F. P., Ozcelik A. O. (2015). Hygiene knowledge of food staff in catering industry. *SAGE Open*.

